# Supplementary material for: The soil microbial community alters patterns of selection on flowering time and fitness‐related traits in Ipomoea purpurea
Source: Am J Bot. 2020 Feb 12;107(2):186–94. doi: 10.1002/ajb2.1426 (PMC7065020; doi:10.1002/ajb2.1426)
Supplement: Supplementary file 1 — APPENDIX S1. Selection differentials (univariate selection analysis; S) for Ipomoea purpurea plant traits. Shown are selection values and model F‐values. Degrees of freedom are listed in parentheses after F‐values. Significant effects are indicated with bold font and asterisks: ***P < 0.001. [file AJB2-107-186-s001.docx]

Chaney and Baucom – American Journal of Botany 2019 – Appendix S1

**APPENDIX S1:** Selection differentials (univariate selection analysis; *S*) for *Ipomoea purpurea* plant traits. Shown are selection values and model *F*-values. Degrees of freedom are listed in parentheses after *F*- values. Significant effects are indicated with bold font and asterisks: ****P* < 0.001.

**Trait** **Selection (*S*)** ***F*-value** Flowering day **-0.22** 36.78(1,192)*** Size **0.14** 14.96(1,192)*** Growth -0.03 0.45(1,192)
