# Supplementary material for: The soil microbial community alters patterns of selection on flowering time and fitness‐related traits in Ipomoea purpurea
Source: Am J Bot. 2020 Feb 12;107(2):186–94. doi: 10.1002/ajb2.1426 (PMC7065020; doi:10.1002/ajb2.1426)
Supplement: Supplementary file 2 — APPENDIX S2. Selection gradients (multivariate selection analysis) for Ipomoea purpurea plant traits. Shown are linear (β) and quadratic (γ) values. Linear coefficients were determined in each treatment from the first‐order model only, whereas the second‐order coefficients were determined from the full model with the linear, squared and cross‐product terms. Quadratic regression coefficients were converted to selection gradients by doubling them. Significant effects are indicated with asterisks: ***P < 0.001. [file AJB2-107-186-s002.docx]

Chaney and Baucom – American Journal of Botany 2019 – Appendix S2

**APPENDIX S2:** Selection gradients (multivariate selection analysis) for *Ipomoea purpurea* plant traits. Shown are linear (*β*) and quadratic (*γ*) values. Linear coefficients were determined in each treatment from the first-order model only, whereas the second-order coefficients were determined from the full model with the linear, squared, and cross-product terms. Quadratic regression coefficients were converted to selection gradients by doubling them. Significant effects are indicated with asterisks: ****P* < 0.001.

| **Trait** | **Linear (*β*)** | **Quadratic (*γ*)** |
| --- | --- | --- |
| Flowering day | -0.20*** | -0.26*** |
| Size | 0.18*** | -0.01 |
| Growth | -0.10*** | -0.02 |
| Flowering day x Size |  | -0.08 |
| Flowering day x Growth |  | -0.04 |
| Size x Growth |  | 0.03 |
