# Supplementary material for: The soil microbial community alters patterns of selection on flowering time and fitness‐related traits in Ipomoea purpurea
Source: Am J Bot. 2020 Feb 12;107(2):186–94. doi: 10.1002/ajb2.1426 (PMC7065020; doi:10.1002/ajb2.1426)

Chaney and Baucom – American Journal of Botany 2019 – Appendix S3

**APPENDIX S3:** Distribution of relative fitness across flowering day in (a) all treatments, (b) complex (autoclave + inoculum) and (c) simple (autoclave) soil treatments. Flowering day is day is standardized to a mean of zero and a variance of one. The high relative fitness values for intermediate traits (mid- to early flowering) indicates stabilizing selection. The line indicates the best fit for quadratic selection for flowering day, shading indicates upper and lower confidence levels.
(a)


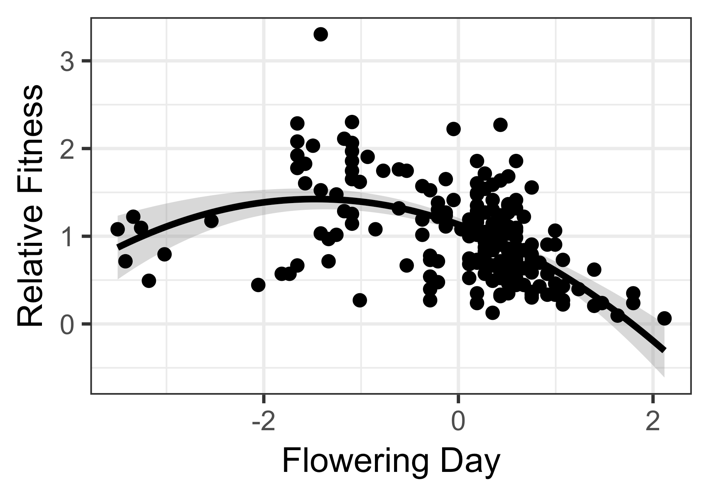


(b) (c)


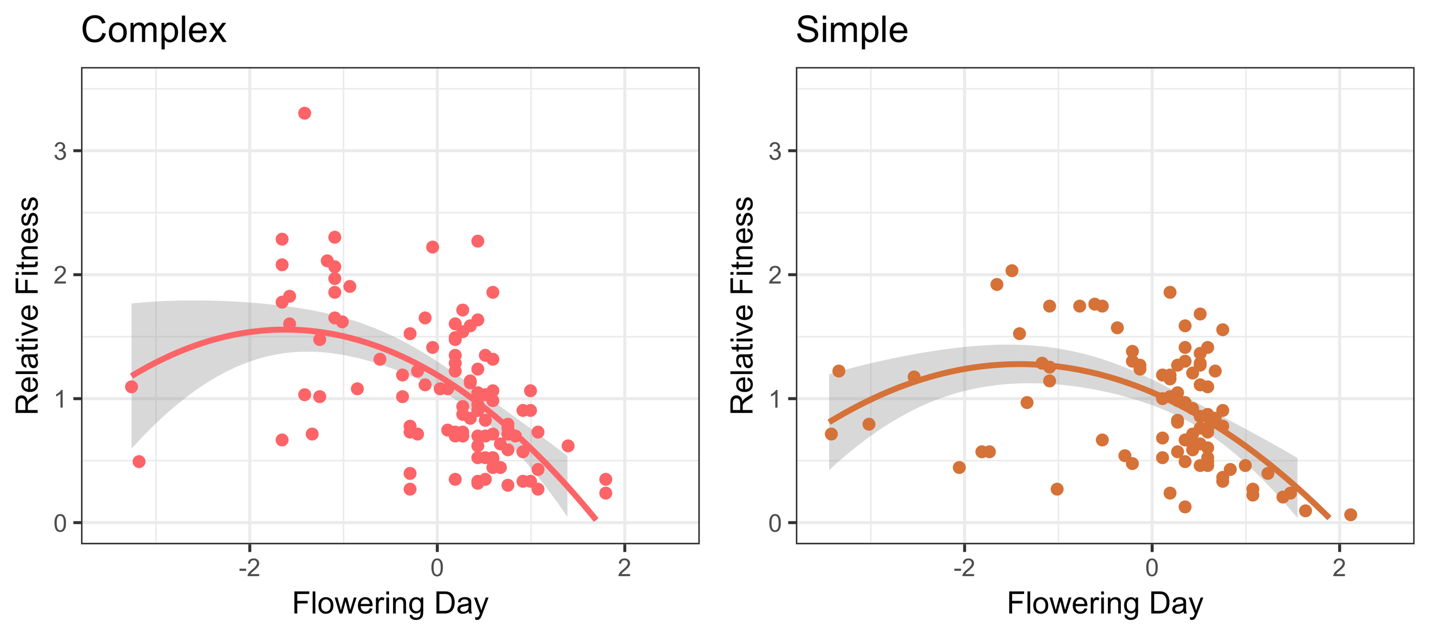

Supplement: Supplementary file 3 — APPENDIX S3. Distribution of relative fitness across flowering day in (a) all treatments, (b) complex (autoclave + inoculum) and (c) simple (autoclave) soil treatments. Flowering day is standardized to a mean of zero and a variance of one. The high relative fitness values for intermediate traits (mid‐ to early flowering) indicates stabilizing selection. The line indicates the best fit for quadratic selection for flowering day, shading indicates upper and lower confidence levels. [file AJB2-107-186-s003.docx]
